# Minimal Products of Coordinate and Momentum Uncertainties of High Orders: Significant and Weak High-Order Squeezing

**DOI:** 10.3390/e22090980

**Published:** 2020-09-03

**Authors:** Miguel Citeli de Freitas, Vitor Dantas Meireles, Viktor V. Dodonov

**Affiliations:** 1 Institute of Physics, University of Brasilia, P.O. Box 04455, Brasilia 70919-970, DF, Brazil; miguelciteli@gmail.com (M.C.d.F.); vitordmeireles@gmail.com (V.D.M.); 2 Instituto de Física de São Carlos, Universidade de São Paulo, C.P. 369, São Carlos 13560-970, SP, Brazil; 3 International Center for Physics, University of Brasilia, Brasilia 70919-970, DF, Brazil

**Keywords:** Fock states, Gaussian states, even/odd coherent states, compass states, generalized intelligent states, minimal uncertainty products, Robertson–Schrödinger uncertainty relations

## Abstract

We consider the problem of minimization of products of mean values of the high powers of operators x and p. From this point of view, we study several two-term superpositions of the Fock states, as well as three popular families of infinite superpositions: squeezed states, even/odd coherent states, and orthogonal even coherent states (or compass states). The new element is the analysis of products of the corresponding (co)variances and the related generalized (Robertson–Schrödinger) intelligent states (RSIS). In particular, we show that both Fock and pure Gaussian homogeneous states are RSIS for the fourth powers (but not for the sixth ones). We show that lower bounds of the high-order uncertainty products can be significantly below the vacuum values. In this connection, the concept of significant and weak high-order squeezing is introduced.

## 1. Introduction

The exact inequality for the product of the second order statistical moments of the dimensionless coordinate and momentum operators (in the system of units with ħ=1),

(1) $$\langle {\hat{x}}^{2}\rangle \langle {\hat{p}}^{2}\rangle \geq 1/4,$$

is known since the paper by Kennard in 1927 [1]. Moreover, it is well known that the equality in (1) is achieved for the vacuum state |0〉 of the quantum harmonic oscillator with $\langle {\hat{x}}^{2}\rangle =\langle {\hat{p}}^{2}\rangle =1/2$. Using the inequality $\langle {\hat{A}}^{2}\rangle \geq {\langle \hat{A}\rangle }^{2}$ (which holds for any Hermitian operator $\hat{A}$) one immediately arrives at the inequality

(2) $$\Pi^{\left(4\right)}\equiv \langle {\hat{x}}^{4}\rangle \langle {\hat{p}}^{4}\rangle \geq 1/16.$$

However, in the vacuum state |0〉, one has $\langle {\hat{x}}^{4}\rangle =3{\langle {\hat{x}}^{2}\rangle }^{2}$ and $\langle {\hat{p}}^{4}\rangle =3{\langle {\hat{p}}^{2}\rangle }^{2}$, so that the left-hand side of (2) equals ${\left(\Pi^{\left(4\right)}\right)}_{vac}=9/16=0.5625$, nine times bigger than the right-hand side! Thus, the natural questions arise: can one find quantum states with the product $\langle {\hat{x}}^{4}\rangle \langle {\hat{p}}^{4}\rangle$ smaller than 9/16 and can the limit value 1/16 be achieved? These questions were considered in [2]. It was shown that the answer to the second question is negative. Using more sophisticated inequalities of the “entropic” type found in [3], the following inequality for the product of statistical moments of the same order can be derived [2]: ${\langle |p|}^{q}{\rangle \langle |x|}^{q}{\rangle \geq \left(\pi e/4\right)}^{q}q^{2(q-1)}e^{-2}{\left[\Gamma \left(1/q\right)\right]}^{-2q}$, where Γ(z) is the Euler gamma function. For q=4, one gets the inequality $\langle {\hat{x}}^{4}\rangle \langle {\hat{p}}^{4}\rangle \geq {\left(4e\pi^{2}\right)}^{2}{\left[\Gamma (1/4)\right]}^{-1/8}\approx 0.3857$. Therefore, the first question becomes: can one find the states with the product $\Pi^{\left(4\right)}$ between 0.39 and 0.56? The positive answer was given in [2], where two simple two-term superpositions of the Fock states of the form

(3) $$|\psi_{k}{\rangle =\alpha |0\rangle +\beta |k\rangle ,\;|\alpha |}^{2}+{\left|\beta \right|}^{2}=1,$$

were considered for k=2 and k=4. It was shown that ${\left(\Pi^{\left(4\right)}\right)}_{min}\approx 0.51$ for $|\psi_{2}\rangle$ (if Im(βα*)=0 and ${\left|\beta \right|}^{2}=1/44$). A smaller value, ${\left(\Pi^{\left(4\right)}\right)}_{min}\approx 0.4901$, was found for $|\psi_{4}\rangle$ with Im(βα*)=0 and Re(βα*)<0, when ${\left|\beta \right|}^{2}=\left(1-\sqrt{150/151}\right)/2\approx 0.00166$ (i.e., |β|≈0.041). These examples show that even a tiny admixture of highly excited states can significantly change the uncertainty product (compared with its value in the vacuum state), due to the quantum interference effects. On the other hand, the overwhelming (but not 100%!) weight of the ground state is very important, since superpositions without the ground state possess high values of $\Pi^{\left(4\right)}$ (see an example in Section 6.1).

Soon after paper [2], Lynch and Mavromatis [4] looked for the minimal value of the product $\Pi^{\left(N\right)}\equiv \langle {\hat{x}}^{N}\rangle \langle {\hat{p}}^{N}\rangle$ in the restricted class of states with equal mean values, $\langle {\hat{x}}^{N}\rangle =\langle {\hat{p}}^{N}\rangle$. They found that the minimal value of $\Pi^{\left(N\right)}$ for such states coincides with the minimal eigenvalue squared $\lambda^{2}$ of the equation

(4) $$\left({\hat{x}}^{N}+{\hat{p}}^{N}\right)|\psi \rangle =2\lambda |\psi \rangle .$$

Using approximate variational schemes, they obtained the minimal value

(5) $${\left(\Pi^{\left(4\right)}\right)}_{LM}={\left(0.6984\right)}^{2}=0.4878=0.8672\;{\left(\Pi^{\left(4\right)}\right)}_{vac}$$

in the specific superposition of the Fock states of the form ${|\psi \rangle }_{K}=\sum_{n=0}^{K}c_{n}|4n\rangle$, with K=6 and certain values of coefficients $c_{n}$ (not given in that papers). Recently, Equation (4) was studied in [5], where the same value $\lambda_{min}^{\left(4\right)}=0.6984$ was confirmed. The value (5) is the best lower limit for the product $\Pi^{\left(4\right)}$ known until now.

An interesting infinite-dimensional analog of the state ${|\psi \rangle }_{K}$ is the superposition of four coherent states with equal amplitudes and phases shifted by π/2,

(6) $${|\psi \rangle }_{4\alpha }=B\left(|\alpha \rangle +|i\alpha \rangle +|-\alpha \rangle +|-i\alpha \rangle \right)=N\sum_{n=0}^{\infty }\frac{\alpha^{4n}}{\sqrt{(4n)!}}|4n\rangle .$$

Such kinds of states were studied for a long time from different points of view, due to their interesting properties. They were named “four-photon states” [6], “orthogonal-even coherent states” [7], “pair cat states” [8], “compass states” [9], and “four-headed cat states” [10]. The state (6) is a special case of large families of “circular states” [11,12,13] and “crystallized cat states” [14]. For extensive lists of references one can consult [15,16]. Following [7], we use the abbreviation OECS for the state (6).

It was shown in [7] that the value ${\left(\Pi^{\left(4\right)}\right)}_{min}\approx 0.49$ can be obtained in the state (6) with |α|≈0.67. Later, this result was confirmed in [17,18]. It is worth noting that the relative probability of the state |4〉 in the superposition (6), $p_{4}/p_{0}={\left(0.67\right)}^{8}/24=0.00169$, is very close to the relative probability 0.00166 of the state |4〉 in the superposition $|\psi_{4}\rangle$ (3).

One of our goals is to analyze some known families of quantum superposition states, in order to see whether they can give the values of $\Pi^{\left(4\right)}$ smaller than 0.49, without the restriction $\langle {\hat{x}}^{N}\rangle =\langle {\hat{p}}^{N}\rangle$. The simplest natural candidates are superpositions of two coherent states or superpositions of states $|\psi_{k}\rangle$ (3), containing even Fock states only. This is explained by the fact that operators ${\hat{x}}^{2}$ and ${\hat{p}}^{2}$ mix the Fock states with the same parity only. Because we need the vacuum state |0〉 as the basic element, only even Fock states can lead to useful interference effects. In principle, one can consider various families of states, which are characterized by arbitrary numbers of independent parameters. However, explicit expressions for the quantity $\Pi^{\left(4\right)}$ are so complicated in the majority of cases that we confine ourselves to the cases of no more than four real parameters.

Another subject of our study is the minimization of the general product $\Pi^{\left(2n\right)}$. Its values in the vacuum state grow very rapidly with increase of power 2n:

(7) $${\left(\Pi^{\left(2n\right)}\right)}_{vac}={\left[2^{-n}\left(2n-1\right)!!\right]}^{2}=2^{-4n}{\left[(2n)!/(n!)\right]}^{2}.$$

In particular, ${\left(\Pi^{\left(4\right)}\right)}_{vac}=9/16=0.5625$,

$${\left(\Pi^{\left(6\right)}\right)}_{vac}=\frac{225}{64}\approx 3.5156,\;{\left(\Pi^{\left(8\right)}\right)}_{vac}=\frac{105^{2}}{256}\approx 43.07,\;{\left(\Pi^{\left(10\right)}\right)}_{vac}=\frac{945^{2}}{32^{2}}\approx 872.1.$$

However, let us look at the ratio $F^{\left(2n\right)}\equiv {\left(\Pi^{\left(2n\right)}\right)}_{min}/{\left(\Pi^{\left(2n\right)}\right)}_{vac}$, where ${\left(\Pi^{\left(2n\right)}\right)}_{min}$ is the minimal value of the product $\Pi^{\left(2n\right)}$. Using the values of ${\left(\Pi^{\left(2n\right)}\right)}_{min}$ calculated in [4] (the best values known up to now), we obtain the following numbers:

$$F^{\left(4\right)}\approx 0.8671,\;F^{\left(6\right)}\approx 0.6201,\;F^{\left(8\right)}\approx 0.40,\;F^{\left(10\right)}\approx 0.37.$$

We see that this ratio diminishes with increase of *n*, but the decrease rate slows down for big values *n*. We study the asymptotic behavior of $F^{\left(2n\right)}$ for the two-Fock superpositions (3) in Section 8, comparing our results with those that were obtained in [4].

The subject that was not addressed in the available literature is the minimal value of the product of variances of operators ${\hat{x}}^{N}$ and ${\hat{p}}^{N}$. Remember that variances and the covariance of two Hermitian operators, $\hat{A}$ and $\hat{B}$, are defined, as follows,

$$\sigma_{A}\equiv \langle {\hat{A}}^{2}\rangle -{\langle \hat{A}\rangle }^{2}\equiv \langle {\left(\delta \hat{A}\right)}^{2}\rangle ,\;\sigma_{AB}\equiv \frac{1}{2}\langle \hat{A}\hat{B}+\hat{B}\hat{A}\rangle -\langle \hat{A}\rangle \langle \hat{B}\rangle \equiv \frac{1}{2}\langle \left\{\delta \hat{A},\delta \hat{B}\right\}\rangle ,\;\delta \hat{A}\equiv \hat{A}-\langle \hat{A}\rangle .$$

Let us define the variance uncertainty product (VUP) of the order 2n as

$$\Pi_{\sigma }^{\left(2n\right)}=\sigma_{x^{n}}\sigma_{p^{n}}\equiv \left(\langle {\hat{x}}^{2n}\rangle -{\langle {\hat{x}}^{n}\rangle }^{2}\right)\left(\langle {\hat{p}}^{2n}\rangle -{\langle {\hat{p}}^{n}\rangle }^{2}\right).$$

Obviously, $\Pi_{\sigma }^{\left(2n\right)}>0$ for any normalized quantum state. It is also obvious that $\Pi_{\sigma }^{\left(2n\right)}\leq \Pi^{\left(2n\right)}$ in any state. However, what is the minimal value of $\Pi_{\sigma }^{\left(2n\right)}$? The answer is well known for n=1: in view of the Heisenberg–Kennard inequality [1] $\sigma_{x}\sigma_{p}\geq 1/4$, one has the minimal value ${\left(\Pi_{\sigma }^{\left(2\right)}\right)}_{min}=1/4$, and this minimum is achieved for all coherent states of the harmonic oscillator. However, the minimum of $\Pi_{\sigma }^{\left(2n\right)}$ with n>1 was never discussed. This issue is considered in Section 2, Section 5 and Section 6 for n=2.

The generalization of inequality $\sigma_{x}\sigma_{p}\geq 1/4$ for arbitrary Hermitian operators $\hat{A}$ and $\hat{B}$ was found by Robertson and Schrödinger [19,20]. It can be written as follows,

(8) $$R\equiv \sigma_{A}\sigma_{B}-\sigma_{AB}^{2}-Y_{AB}^{2}\geq 0,\;Y_{AB}=\frac{1}{2i}\langle [\hat{A},\hat{B}]\rangle .$$

Two equivalent formulas for *R*, which can be useful in some cases, are

$$R\equiv \langle {\left(\delta \hat{A}\right)}^{2}\rangle \langle {\left(\delta \hat{B}\right)}^{2}\rangle -{\left|\langle \left(\delta \hat{A}\right)\left(\delta \hat{B}\right)\rangle \right|}^{2},$$

$$R\equiv \langle {\hat{A}}^{2}\rangle \langle {\hat{B}}^{2}\rangle -{\left|\langle \hat{A}\hat{B}\rangle \right|}^{2}-\langle {\hat{A}}^{2}\rangle {\langle \hat{B}\rangle }^{2}-\langle {\hat{B}}^{2}\rangle {\langle \hat{A}\rangle }^{2}+\langle \hat{A}\hat{B}+\hat{B}\hat{A}\rangle \langle \hat{A}\rangle \langle \hat{B}\rangle .$$

The problem with inequality (8) consists in the fact that the quantity $Y_{AB}$ is state dependent for many operators, different from $\hat{x}$ and $\hat{p}$. Moreover, $Y_{AB}$ can be equal to zero in many quantum states. Therefore, the following non-negative quantities can be studied in connection with inequality (8):

(9) $$D\equiv \sigma_{A}\sigma_{B}-\sigma_{AB}^{2},\;Z\equiv \sigma_{A}\sigma_{B}-Y_{AB}^{2}.$$

The name “intelligent states” (IS) was coined in [21] for the spin (angular momentum) states satisfying the equality Z=0. The difference between “intelligent” and “minimum uncertainty” states for the angular momentum operators was discussed in [22]. A little later, the same name was accepted in connection with the non-compact group SU(1,1) [23]. Further extensions of the concept of “intelligent” states were made, e.g., in [24,25,26,27]. Of course, the word “intelligent” is nothing but a label, but this name has been adopted nowadays by the majority of researchers. Later on, the states satisfying a more general equality R=0 were named “generalized intelligent states” (GIS) in [28]. The names “Schrödinger intelligent states” and “Robertson intelligent states” were also used in [29,30]. Many references to the papers that are devoted to IS and GIS for various sets of operators can be found in [15]. The physical meaning of IS and methods of their generation were discussed, e.g., in papers [31,32,33,34]. “Constrained” intelligent states were introduced in study [35]. We shall use the name “ordinary intelligent state” (OIS) in the case of Z=0, and the name “Robertson–Schrödinger intelligent state” (RSIS) when R=0.

In Section 2, we consider the operators $\hat{A}={\hat{x}}^{N}$ and $\hat{B}={\hat{p}}^{N}$ and Gaussian pure (squeezed) states, trying to answer the following questions: (1) When the equalities R=0 or Z=0 can be satisfied? (2) What is the minimal value of the quantity *D*? In Section 3, we show that the Fock states are RSIS (but not OIS) for N=2. Section 4, Section 5, Section 6, Section 7 and Section 8 are devoted to the detailed analysis of the products $\Pi^{\left(2n\right)}$ and $\Pi_{\sigma }^{\left(2n\right)}$ in various superposition states, described by a small number (from 2 to 4) of real parameters. The main goal is to minimize these products with respect to the chosen parameters. The results of that sections naturally lead us to some thoughts about the best definition of the concept of the high-order squeezing. This subject is discussed in Section 9. The last Section 10 contains a brief discussion of main results that were obtained in the paper, together with outlines of possible directions for further studies.

## 2. Robertson–Schrödinger Relations with High-Order Moments for the Gaussian States

It is known since [1] that R≡0 for A=x and B=p in any pure Gaussian state, described by means of the normalized wave function

(10) $$\psi \left(x\right)={\left(\frac{a}{\pi }\right)}^{1/4}\exp\left[-\frac{1}{2}\left(a+ib\right)x^{2}+\left(c+id\right)x-\frac{c^{2}}{2a}\right],$$

where a,b,c,d are real numbers with a>0. If b=0 and a=1, then (10) is the well known Schrödinger’s non-dispersive wave packet, called nowadays the Glauber–Sudarshan coherent state. The states with arbitrary *a* and b≠0 were named “correlated coherent states” in [36]. The states with c=d=0 are nothing but the “squeezed vacuum states”. For a detailed history of these states, one can consult review [15].

Coefficients of the wave function (10) can be written in terms of mean values and variances:

(11) $$a={\left(2\sigma_{x}\right)}^{-1},\;c=\langle \hat{x}\rangle /\left(2\sigma_{x}\right),\;b=-\sigma_{xp}/\sigma_{x},\;d=\langle \hat{p}\rangle -\langle \hat{x}\rangle \sigma_{xp}/\sigma_{x}.$$

The high-order moments of operators $\hat{x}$ and $\hat{p}$ in the state (10) are given by the formulas (see Appendix A for the proof)

(12) $$\langle {\hat{x}}^{n}\rangle =i^{-n}{\left(\sigma_{x}/2\right)}^{n/2}H_{n}\left(i\langle \hat{x}\rangle /\sqrt{2\sigma_{x}}\right),\;\langle {\hat{p}}^{n}\rangle =i^{-n}{\left(\sigma_{p}/2\right)}^{n/2}H_{n}\left(i\langle \hat{p}\rangle /\sqrt{2\sigma_{p}}\right),$$

where $H_{n}\left(z\right)$ is the Hermite polynomial. The right-hand sides of expressions in (12) are always real, due to the even/odd parity of the Hermite polynomials for even/odd values of power *n*. If $\langle \hat{x}\rangle =\langle \hat{p}\rangle =0$, then

(13) $$\langle {\hat{x}}^{2n}\rangle =\frac{(2n)!}{2^{n}n!{\left(2a\right)}^{n}}=\frac{(2n-1)!!}{{\left(2a\right)}^{n}},\;\langle {\hat{p}}^{2n}\rangle =\frac{(2n-1)!!}{{\left(2a\right)}^{n}}{\left(a^{2}+b^{2}\right)}^{n}.$$

A simple formula can also be obtained for the mean value $\langle {\hat{x}}^{n}{\hat{p}}^{n}\rangle$, if $\langle \hat{x}\rangle =\langle \hat{p}\rangle =0$:

(14) $$\langle {\hat{x}}^{n}{\hat{p}}^{n}\rangle ={\langle {\hat{p}}^{n}{\hat{x}}^{n}\rangle }^{*}=i^{n}n!{\left[\left(a+ib\right)/\left(2a\right)\right]}^{n/2}P_{n}\left(\sqrt{\left(a+ib)/(2a\right)}\right),$$

where $P_{n}\left(z\right)$ is the Legendre polynomial. The consequence of (14) for n=1 is the known formula for the covariance in the Gaussian state (10): $\sigma_{xp}=-b/\left(2a\right)$.

### 2.1. N = 2

For $\hat{A}={\hat{x}}^{2}$ and $\hat{B}={\hat{p}}^{2}$, we have

$$\langle {\hat{A}}^{2}\rangle =\langle {\hat{x}}^{4}\rangle =\frac{3}{4a^{2}}+\frac{3c^{2}}{a^{3}}+\frac{c^{4}}{a^{4}},\;\langle \hat{A}\rangle =\langle {\hat{x}}^{2}\rangle =\frac{1}{2a}+\frac{c^{2}}{a^{2}},\;\sigma_{A}=\frac{1}{2a^{2}}+\frac{2c^{2}}{a^{3}}=2\sigma_{x}^{2}+4\sigma_{x}{\langle \hat{x}\rangle }^{2}.$$

In view of the x−p symmetry, we can write

$$\sigma_{B}\equiv \langle {\hat{p}}^{4}\rangle -{\langle {\hat{p}}^{2}\rangle }^{2}=2\sigma_{p}^{2}+4\sigma_{p}{\langle \hat{p}\rangle }^{2}={\left(a^{2}+b^{2}\right)}^{2}/\left(2a^{2}\right)+2\left(a^{2}+b^{2}\right){\left(ad-bc\right)}^{2}/a^{3}.$$

Therefore, the minimum of $\Pi_{\sigma }^{\left(4\right)}$ is achieved for the states with b=c=d=0, i.e., for all uncorrelated vacuum squeezed states (here, we restore the Planck constant):

(15) $${\left(\Pi_{\sigma }^{\left(4\right)}\right)}_{min}={\left(\Pi_{\sigma }^{\left(4\right)}\right)}_{Gauss}=\hbar^{4}/4=\hbar^{2}{\left(\Pi_{\sigma }^{\left(2\right)}\right)}_{min}.$$

In addition,

(16) $$\langle {\hat{x}}^{2}{\hat{p}}^{2}\rangle =\left(\alpha -\beta^{2}\right)\left[{\langle \hat{x}\rangle }^{2}+\sigma_{x}\right]+2\alpha \beta \left[{\langle \hat{x}\rangle }^{3}+3\langle \hat{x}\rangle \sigma_{x}\right]-\alpha^{2}\left[{\langle \hat{x}\rangle }^{4}+6{\langle \hat{x}\rangle }^{2}\sigma_{x}+3\sigma_{x}^{2}\right],$$

where α=a+ib and β=c+id. Using the real part of (16), together with (A4), we obtain a very simple formula for the covariance: $\sigma_{AB}\equiv \langle {\hat{x}}^{2}{\hat{p}}^{2}+{\hat{p}}^{2}{\hat{x}}^{2}\rangle /2-\langle {\hat{x}}^{2}\rangle \langle {\hat{p}}^{2}\rangle =4\langle \hat{x}\rangle \langle \hat{p}\rangle \sigma_{xp}+2\sigma_{xp}^{2}-1/2$. Consequently, $D_{AB}=4{\left(\sigma_{xp}+\langle \hat{x}\rangle \langle \hat{p}\rangle \right)}^{2}+8\sigma_{x}\sigma_{p}\left(\sigma_{x}{\langle \hat{p}\rangle }^{2}+\sigma_{p}{\langle \hat{x}\rangle }^{2}-2\sigma_{xp}\langle \hat{x}\rangle \langle \hat{p}\rangle \right)$, so that $D_{AB}^{\left(min\right)}=0$ for b=c=d=0.

Because $\left[{\hat{x}}^{2},{\hat{p}}^{2}\right]=2i\left(\hat{x}\hat{p}+\hat{p}\hat{x}\right)$, we immediately obtain the value $Y_{AB}=2\left(\sigma_{xp}+\langle \hat{x}\rangle \langle \hat{p}\rangle \right)$. Therefore,

$$R=8\sigma_{x}\sigma_{p}\left(\sigma_{x}{\langle \hat{p}\rangle }^{2}+\sigma_{p}{\langle \hat{x}\rangle }^{2}-2\sigma_{xp}\langle \hat{x}\rangle \langle \hat{p}\rangle \right)=\left(a^{2}+b^{2}\right)\left(c^{2}+d^{2}\right)/a^{3}.$$

This quantity equals zero for c=d=0 and arbitrary values of *a* and *b*. Consequently, any squeezed vacuum state is the Robertson–Schrödinger intelligent state not only for the pair of operators ($\hat{x},\hat{p}$), but also for the pair (${\hat{x}}^{2},{\hat{p}}^{2}$). Actually, this fact can be derived from the results of study [28], since linear combinations of operators ${\hat{x}}^{2}$, ${\hat{p}}^{2}$ and $\hat{x}\hat{p}+\hat{p}\hat{x}$ are generators of the su(1,1) algebra. The new result obtained here is that the presence of linear terms in the argument of the exponential function (10) breaks the fourth-order generalized “intelligence”, in contradistinction to the second-order “intelligence”.

Another interesting formula is

$$Z\equiv \sigma_{A}\sigma_{B}-Y_{AB}^{2}=4\left(\sigma_{x}^{2}\sigma_{p}^{2}-\sigma_{xp}^{2}\right)+4{\langle \hat{x}\rangle }^{2}{\langle \hat{p}\rangle }^{2}\left(4\sigma_{x}\sigma_{p}-1\right)+8\sigma_{x}\sigma_{p}\left(\sigma_{x}{\langle \hat{p}\rangle }^{2}+\sigma_{p}{\langle \hat{x}\rangle }^{2}\right).$$

The second and third terms in the right-hand side are always non-negative, going to zero simultaneously for $\langle \hat{x}\rangle =\langle \hat{p}\rangle =0$ only. On the other hand, $\sigma_{x}^{2}\sigma_{p}^{2}-\sigma_{xp}^{2}={\left(a^{2}-b^{2}\right)}^{2}/\left(16a^{4}\right)$. Consequently, the correlated state (10) is the OIS for the pair (${\hat{x}}^{2},{\hat{p}}^{2}$), if c=d=0 and b=±a, when $\sigma_{xp}=\mp 1/2$ and the correlation coefficient $r\equiv \sigma_{xp}/\sqrt{\sigma_{x}\sigma_{p}}=\mp 1/\sqrt{2}$. This result is new. Note that $\sigma_{AB}=0$, in this case, so that the state is uncorrelated from the point of view of the (${\hat{x}}^{2},{\hat{p}}^{2}$) pair.

### 2.2. N = 3, Homogeneous States

For $\hat{A}={\hat{x}}^{3}$ and $\hat{B}={\hat{p}}^{3}$, we consider the state (10) without linear terms (c=d=0). Subsequently, $\langle \hat{x}\rangle =\langle {\hat{x}}^{3}\rangle =\langle \hat{p}\rangle =\langle {\hat{p}}^{3}\rangle =0$, so that

$$\sigma_{A}=\frac{15}{8a^{3}},\;\sigma_{B}=\frac{15a^{3}}{8}{\left(1+g^{2}\right)}^{3},\;\sigma_{AB}=\frac{3}{8}\left(3g-5g^{3}\right),\;Y_{AB}=\frac{3}{8}\left(1+9g^{2}\right),\;g\equiv b/a.$$

Consequently, $\Pi_{\sigma }^{\left(6\right)}=\Pi^{\left(6\right)}=\left(225/64\right){\left(1+g^{2}\right)}^{3}$ and $D=(225+594g^{2}+945g^{4})/64$. Two other quantities, $R=27{\left(1+g^{2}\right)}^{2}/8$ and $Z=(216+513g^{2}-54g^{4}+225g^{6})/64$, attain minimal values at g=0. Therefore, Gaussian states cannot be “intelligent” in any sense for the pair (${\hat{x}}^{3},{\hat{p}}^{3}$).

### 2.3. Arbitrary N, Vacuum Squeezed States

For the vacuum squeezed (uncorrelated) states (b=c=d=0), we obtain, from (13), the uncertainty product of the 2nth order, as given by Equation (7). The same value corresponds to $\Pi_{\sigma }^{\left(2n\right)}$, if *n* is any odd number. If n=2m, then

$$\Pi_{\sigma }^{\left(4m\right)}=2^{-4m}{\left\{\left(4m-1\right)!!-{\left[\left(2m-1\right)!!\right]}^{2}\right\}}^{2}=2^{-8m}{\left\{\frac{(4m)!}{(2m)!}-{\left[\frac{(2m)!}{m!}\right]}^{2}\right\}}^{2}.$$

However, using the known Stirling formula for n≫1, $n!\approx \sqrt{2\pi n}{\left(n/e\right)}^{n}$, one can see that the relative contribution of the second term inside the figure brackets rapidly decreases with *m* as $4^{-m}\sqrt{2}$. Consequently, we can neglect the difference between $\Pi^{\left(2n\right)}$ and $\Pi_{\sigma }^{\left(2n\right)}$ for n≫1, using the approximate formula $\Pi^{\left(2n\right)}\approx 2{\left(n/e\right)}^{2n}$, independently of the parity of number *n*. Moreover, Formula (14), together with the known inequality $|P_{n}\left(\cos\phi \right)|\leq 1$, shows that $R∼Z∼D∼\Pi^{\left(2n\right)}$, if n≫1.

## 3. Fock States as Intelligent States

Simple formulas can be obtained for various combinations of products of the second-order moments of operators $\hat{A}={\hat{x}}^{2}$ and $\hat{B}={\hat{p}}^{2}$ in an arbitrary Fock state |n〉:

$$\langle {\hat{x}}^{2}\rangle =\langle {\hat{p}}^{2}\rangle =n+1/2,\;\langle {\hat{x}}^{4}\rangle =\langle {\hat{p}}^{4}\rangle =\frac{1}{4}\left(6n^{2}+6n+3\right),\;\langle {\hat{x}}^{2}{\hat{p}}^{2}\rangle =\frac{1}{4}\left(2n^{2}+2n-1\right).$$

They follow from the standard expressions for the canonical dimensionless operators $\hat{x}$ and $\hat{p}$ in terms of the bosonic annihilation and creation operators,

(17) $$\hat{x}=\left(a+a^{†}\right)/\sqrt{2},\;\hat{p}=\left(a-a^{†}\right)/\left(i\sqrt{2}\right),\;\hat{a}|n\rangle =\sqrt{n}|n-1\rangle ,\;{\hat{a}}^{†}|n\rangle =\sqrt{n+1}|n+1\rangle .$$

Consequently,

$$\sigma_{A}=\sigma_{B}=\frac{1}{2}\left(n^{2}+n+1\right),\;\sigma_{AB}=-\;\frac{1}{2}\left(n^{2}+n+1\right).$$

In addition, $Y_{AB}=0$, as soon as $\langle \hat{x}\hat{p}+\hat{p}\hat{x}\rangle =0$. Therefore, D=R=0 for any Fock state. This means that all of the Fock states are Robertson–Schrödinger intelligent states for the pair of operators $\hat{A}={\hat{x}}^{2}$ and $\hat{B}={\hat{p}}^{2}$. However, they are not ordinary intelligent states for this pair. The product $\sigma_{A}\sigma_{B}$ equals 1/4 for n=0, and it grows rapidly with increase of *n*. However, we can expect that some superpositions of the vacuum and a few first excited states can give smaller values of this product. This conjecture is analyzed in the next sections.

## 4. Nth Order Products in the Two-Term Even Fock Superpositions: General Formulas

In this section, we consider the mean values of even powers of the coordinate and momentum in the two-term superpositions of the even Fock states $|\psi_{2j}\rangle$ (3). In this case, we can write

(18) 〈ψ2j|x^2n|ψ2j〉=|α|2μ(2n)+|β|2ν2j(2n)+2Re(α*β)ρ2j(2n)≡μ(2n)1+|β|2ν˜2j(2n)+2Re(α*β)ρ˜2j(2n),

$$\mu^{\left(2n\right)}=\langle 0|{\hat{x}}^{2n}|0\rangle =2^{-n}\left(2n-1\right)!!=2^{-2n}\left(2n\right)!/n!,$$

(19) $$\nu_{2j}^{\left(2n\right)}=\langle 2j|{\hat{x}}^{2n}|2j\rangle ,\;\rho_{2j}^{\left(2n\right)}=\langle 0|{\hat{x}}^{2n}|2j\rangle ,\;{\tilde{\nu }}_{2j}^{\left(2n\right)}=\nu_{2j}^{\left(2n\right)}/\mu^{\left(2n\right)}-1,\;{\tilde{\rho }}_{2j}^{\left(2n\right)}=\rho_{2j}^{\left(2n\right)}/\mu^{\left(2n\right)}.$$

All of the coefficients (19) are real non-negative numbers. This is the consequence of formulas (17). Besides, $\rho_{2j}^{\left(2n\right)}=0$ if j>n. Using the form of wave functions in the coordinate and momentum representations,

$$\langle x|n\rangle ={\left(\sqrt{\pi }2^{n}n!\right)}^{-1/2}H_{n}\left(x\right)e^{-x^{2}/2},\;\langle p|n\rangle ={\left(-i\right)}^{n}{\left(\sqrt{\pi }2^{n}n!\right)}^{-1/2}H_{n}\left(p\right)e^{-x^{2}/2},$$

we obtain the formula

(20) 〈ψ2j|p^2n|ψ2j〉=|α|2μ(2n)+|β|2ν2j(2n)+2(−1)jRe(α*β)ρ2j(2n),

with the same coefficients as in (18).

According to Equation (18), the minimum of $\langle \psi_{2j}|{\hat{x}}^{2n}|\psi_{2j}\rangle$ with a fixed value of |β| is achieved for Re(α*β)=−|αβ|. In order to find the absolute minimum, we have to minimize the function

(21) $$f\left(z\right)=1+\tilde{\nu }z-2\tilde{\rho }\sqrt{z(1-z)},\;z={\left|\beta \right|}^{2}.$$

Here, we dropped all indexes. Calculating the derivative df/dz, we arrive at the quadratic equation $g^{2}z^{2}-g^{2}z+\rho^{2}=0$, where $g^{2}=4{\tilde{\rho }}^{2}+{\tilde{\nu }}^{2}$. It has two real solutions. We need the solution $z_{*}$, which is close to zero. Afterwards,

(22) $$z_{*}=\frac{g-\tilde{\nu }}{2g}=\frac{2{\tilde{\rho }}^{2}}{g(g+\tilde{\nu })},\;f_{min}=1-\frac{g-\tilde{\nu }}{2}=1-\frac{2{\tilde{\rho }}^{2}}{g+\tilde{\nu }},\;g=\sqrt{4{\tilde{\rho }}^{2}+{\tilde{\nu }}^{2}}.$$

These formulas are exact. For small values of |β| (which are most relevant for our purposes, as will be shown in the following sections), one can use the parabolic approximation $f\left(|\beta |\right)=1+\tilde{\nu }{\left|\beta \right|}^{2}-2\tilde{\rho }\left|\beta \right|$, which results in simple formulas ${\left|\beta \right|}_{min}=\tilde{\rho }/\tilde{\nu }$ and $f_{min}=1-{\tilde{\rho }}^{2}/\tilde{\nu }$.

If *j* is odd, then the minimum of $\langle \psi_{2j}|{\hat{x}}^{2n}|\psi_{2j}\rangle$ corresponds to the maximum of $\langle \psi_{2j}|{\hat{p}}^{2n}|\psi_{2j}\rangle$, according to Equations (18) and (20). In this case, ${\left(\Pi^{\left(2n\right)}\right)}_{min}=\chi_{min}{\left(\Pi^{\left(2n\right)}\right)}_{vac}$, where $\chi_{min}$ is the minimum of function $\chi \left(z\right)={\left(1+\tilde{\nu }z\right)}^{2}-4{\tilde{\rho }}^{2}z\left(1-z\right)$. The exact position of minimum $z_{*}$ and exact minimal value $\chi_{min}$ are as follows:

(23) $$z_{*}=\frac{2{\tilde{\rho }}^{2}-\tilde{\nu }}{{\tilde{\nu }}^{2}+4{\tilde{\rho }}^{2}},\;\chi_{min}=\frac{4{\tilde{\rho }}^{2}\left(1+\tilde{\nu }-{\tilde{\rho }}^{2}\right)}{{\tilde{\nu }}^{2}+4{\tilde{\rho }}^{2}}.$$

Explicit expressions for the coefficients (19) in the most general case are as follows (see Appendix B):

(24) $${\tilde{\rho }}_{2j}^{\left(2n\right)}=\frac{2^{j}n!}{\left(n-j\right)!\sqrt{(2j)!}}=\frac{2^{j}}{\sqrt{(2j)!}}\left(n-j+1\right)\left(n-j+2\right)\cdots n,$$

(25) $${\tilde{\nu }}_{2j}^{\left(2n\right)}+1=\frac{2^{2j}}{(2j)!}\left(n+1\right)\left(n+2\right)\cdots \left(n+2j\right)F\left(-2j,-2j;-n-2j;1/2\right),$$

where F(a,b;c;z) is the Gauss hypergeometric function.

## 5. Fourth-Order Variance Products in States $|\psi_{2}\rangle$ and $|\psi_{4}\rangle$

General formulas for the variances $\sigma_{A}$ and $\sigma_{B}$ are rather involved for arbitrary numbers *n* and *j*. In this section, we consider in detail the case of n=2, when all calculations can be performed in a relatively simple way.

### 5.1. Superpositions of the Vacuum and Fourth Fock States

For n=j=2, the general scheme of the preceding section results in the following mean values:

〈x^2〉=〈p^2〉=12+4|β|2,〈x^4〉=〈p^4〉=34+30|β|2+6Re(α*β).

Therefore,

(26) σA=σB=12+6Re(α*β)+26|β|2−16|β|4.

For the fixed values of |α| and |β|, the minimum of (26) is achieved for Re(α*β)=−|αβ|. Thus, we arrive at the function

$$f\left(|\beta |\right)=\frac{1}{2}-\left|\beta \right|\sqrt{6(1-|\beta {|}^{2})}+{26|\beta |}^{2}-16{\left|\beta \right|}^{4}.$$

Its minimum in the interval 0<|β|<1 can be found numerically:

(27) $$\sigma_{A}^{\left(min\right)}=\sigma_{B}^{\left(min\right)}\approx 0.4424,\;{\left(\Pi_{\sigma }^{\left(4\right)}\right)}_{min}\approx {0.1957,\;|\beta |}_{min}\approx 0.0471,\;{\left|\beta \right|}_{min}^{2}\approx 0.00222.$$

### 5.2. Superpositions of the Vacuum and Second Fock States

For n=2 and j=1, we have unequal second- and fourth-order moments:

〈x^2〉=12+2|β|2+2Re(α*β),〈p^2〉=12+2|β|2−2Re(α*β),

〈x^4〉=34+9|β|2+32Re(α*β),〈p^4〉=34+9|β|2−32Re(α*β),

σA=12+7|β|2+22Re(α*β)−22|β|2+Re(α*β)2,

σB=12+7|β|2−22Re(α*β)−22|β|2−Re(α*β)2,

Πσ(4)=148|β|4−14|β|2−12−2[Re(α*β)]28|β|4−2|β|2+5+4[Re(α*β)]4.

Writing Re(α*β)=|αβ|cos(ϕ), one can see that $\Pi_{\sigma }^{\left(4\right)}$ is a monotonous function of $\cos^{2}\left(\phi \right)$, with the minimum at $\cos^{2}\left(\phi \right)=1$. Therefore, writing [Re(α*β)]2=|β|2(1−|β|2), we arrive at the function

$$\Pi_{\sigma }^{\left(4\right)}={36|\beta |}^{8}-{84|\beta |}^{6}+{63|\beta |}^{4}-3{\left|\beta \right|}^{2}+1/4,$$

which attains the minimum (found numerically)

$${\left(\Pi_{\sigma }^{\left(4\right)}\right)}_{min}\approx 0.213076\;\;\mathrm{at}\;|\beta |\approx {0.15826,\;|\beta |}^{2}\approx 1/40.$$

For this value of |β| and cos(ϕ)=1, we have

$$\langle {\hat{x}}^{2}\rangle =0.771085,\;\langle {\hat{x}}^{4}\rangle =1.638394,\;\sigma_{A}=1.043822,$$

$$\langle {\hat{p}}^{2}\rangle =0.329099,\;\langle {\hat{p}}^{4}\rangle =0.312438;,\;\sigma_{B}=0.204131.$$

Minimal values of $\langle {\hat{p}}^{2}\rangle$, $\langle {\hat{p}}^{4}\rangle$ and $\sigma_{B}$ are obtained for cos(ϕ)=1. They are as follows,

$${\langle {\hat{p}}^{2}\rangle }_{min}=0.275255\;\;\mathrm{for}\;|\beta |=0.302905,\;{\langle {\hat{p}}^{4}\rangle }_{min}=0.275063\;\;\mathrm{for}\;\left|\beta \right|=0.218479,$$

$$\;\sigma_{B}^{{}_{min}}=0.186765\;\;\mathrm{for}\;\left|\beta \right|=0.203724.$$

**Figure 1:**
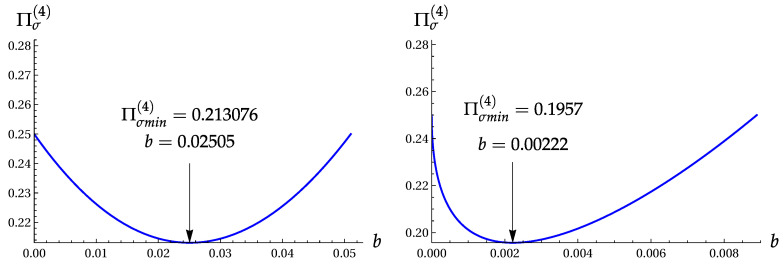
The product $\Pi_{\sigma }^{\left(4\right)}$ versus $b={\left|\beta \right|}^{2}$ for the two-term Fock superpositions $|\psi_{k}\rangle$ (3). Left: for $|\psi_{2}\rangle$ with $\cos^{2}\left(\phi \right)=1$. Right: for $|\psi_{4}\rangle$ with cos(ϕ)=−1.

Figure 1 shows the product $\Pi_{\sigma }^{\left(4\right)}$ as function of $b={\left|\beta \right|}^{2}$ for the superpositions $|\psi_{2}\rangle$ and $|\psi_{4}\rangle$ (3).

**Figure 2:**
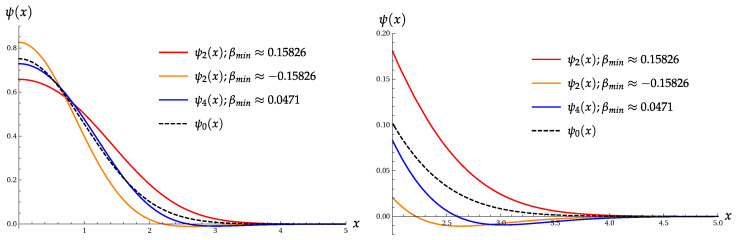
Wave functions $\psi_{2}\left(x\right)$ and $\psi_{4}\left(x\right)$ with the extremal values of parameter β, as compared with the ground-state wave function $\psi_{0}\left(x\right)$.

Figure 2 shows the wave functions $\psi_{2}\left(x\right)$ and $\psi_{4}\left(x\right)$ with the extremal values of parameter β, as compared with the ground-state wave function $\psi_{0}\left(x\right)$.

Note that two functions with β=±0.15826 have the same value of the product $\Pi_{\sigma }^{\left(4\right)}$, but they have different values of $\sigma_{A}$ and $\sigma_{B}$.

### 5.3. Superpositions of Three Even Fock States

Now, let us consider the three-parameter states of the form

$$|\psi \rangle =C_{0}|0\rangle +C_{1}|2\rangle e^{i\phi }+C_{2}{|4\rangle }^{i\chi },\;C_{0}^{2}+C_{1}^{2}+C_{2}^{2}=1,$$

where all three coefficients $C_{j}$ can be assumed to be real and non-negative. General formulas in this case are rather involved. For example,

$$\langle {\hat{x}}^{4}\rangle =3/4+9C_{1}^{2}+30C_{2}^{2}+\sqrt{6}C_{0}C_{2}\cos\left(\chi \right)+3\sqrt{2}C_{0}C_{1}\cos\left(\phi \right)+14\sqrt{3}C_{1}C_{2}\cos\left(\chi -\phi \right),$$

$$\langle {\hat{p}}^{4}\rangle =3/4+9C_{1}^{2}+30C_{2}^{2}+\sqrt{6}C_{0}C_{2}\cos\left(\chi \right)-3\sqrt{2}C_{0}C_{1}\cos\left(\phi \right)-14\sqrt{3}C_{1}C_{2}\cos\left(\chi -\phi \right).$$

If $C_{1}=0$, then both products, $\Pi^{\left(4\right)}$ or $\Pi_{\sigma }^{\left(4\right)}$, attain their minimal values for χ=π. Using this value, we can write $\Pi^{\left(4\right)}$ as $\Pi^{\left(4\right)}={\left(3/4+9C_{1}^{2}+30C_{2}^{2}-\sqrt{6}C_{0}C_{2}\right)}^{2}-C_{1}^{2}\cos^{2}\left(\phi \right){\left(3\sqrt{2}C_{0}-14\sqrt{3}C_{2}\right)}^{2}$. The partial derivative of this function with respect to $C_{1}^{2}$ at $C_{1}^{2}=0$ equals

$${\left.\partial \Pi^{\left(4\right)}/\partial C_{1}^{2}\right|}_{C_{1}^{2}=0}=\left(3/4+30C_{2}^{2}-\sqrt{6}C_{0}C_{2}\right)\left(18+\sqrt{6}C_{2}/C_{0}\right)-\cos^{2}\left(\phi \right){\left(3\sqrt{2}C_{0}-14\sqrt{3}C_{2}\right)}^{2}.$$

Using the value $C_{2}=0.041$ (when function $\Pi^{\left(4\right)}\left(C_{2},C_{1}=0\right)$ attains the minimum), we obtain ${\left.\partial \Pi^{\left(4\right)}/\partial C_{1}^{2}\right|}_{C_{1}^{2}=0}\approx 12.7-10.5\cos^{2}\left(\phi \right)$. This derivative is positive for any phase ϕ. Consequently, the addition of the second Fock state |2〉 can only spoil the result obtained for the two-term superposition $|\psi_{4}\rangle$. The same conclusion holds for the product $\Pi_{\sigma }^{\left(4\right)}$ (although the calculations are more cumbersome).

## 6. Superpositions of Coherent States

### 6.1. Two Coherent States

The first simplest superpositions of two coherent states were introduced in [37] under the names even/odd coherent states:

(28) $${|\alpha \rangle }_{\pm }=N_{\pm }\left(|\alpha \rangle \pm |-\alpha \rangle \right),\;N_{\pm }^{-2}=2\left[1\pm \exp\left(-{2|\alpha |}^{2}\right)\right].$$

Here, |α〉 is the well known Glauber–Sudarshan coherent state, satisfying the equation $\hat{a}|\alpha \rangle =\alpha |\alpha \rangle$ with $\hat{a}=\left(\hat{x}+i\hat{p}\right)/\sqrt{2}$. Remember that wave functions of the coherent states in the coordinate and momentum representations have the form

(29) $$\langle x|\alpha \rangle =\pi^{-1/4}\exp\left(-\frac{x^{2}}{2}+\sqrt{2}\alpha x-\frac{\alpha^{2}}{2}-\frac{{\left|\alpha \right|}^{2}}{2}\right),\;\;\langle p|\alpha \rangle =\pi^{-1/4}\exp\left(-\frac{p^{2}}{2}-i\sqrt{2}\alpha p+\frac{\alpha^{2}}{2}-\frac{{\left|\alpha \right|}^{2}}{2}\right).$$

Therefore,

(30) $${\langle x|\alpha \rangle }_{+}=\frac{\pi^{-1/4}\cosh\left(\sqrt{2}\alpha x\right)}{\sqrt{\cosh(|\alpha {|}^{2})}}\exp\left(-\frac{x^{2}}{2}-\frac{\alpha^{2}}{2}\right),\;{\langle p|\alpha \rangle }_{+}=\frac{\pi^{-1/4}\cos\left(\sqrt{2}\alpha p\right)}{\sqrt{\cosh(|\alpha {|}^{2})}}\exp\left(-\frac{p^{2}}{2}+\frac{\alpha^{2}}{2}\right).$$

Note that ${|0\rangle }_{+}$ is the ground Fock state |0〉, whereas ${|0\rangle }_{-}$ is the first excited Fock state |1〉. The states (28) and their generalizations were discussed in detail, e.g., in reviews [15,38,39]. For our purposes, the following properties of the states (28) are useful:

$${}_{-}{\langle \alpha |\alpha \rangle }_{+}=0,\;\hat{a}{|\alpha \rangle }_{\pm }=\alpha \left(N_{\mp }/N_{\pm }\right){|\alpha \rangle }_{\mp },\;{\left(N_{+}/N_{-}\right)}^{2}={\tanh(|\alpha |}^{2}).$$

Using these properties, the following mean values can be found:

$${\langle {\hat{x}}^{2}\rangle }_{\pm }=1/2+at_{\pm }+ac_{2},\;{\langle {\hat{p}}^{2}\rangle }_{\pm }=1/2+at_{\pm }-ac_{2},$$

$${\langle {\hat{x}}^{4}\rangle }_{\pm }=\frac{3}{4}+a^{2}+a^{2}c_{2}^{2}+3at_{\pm }+ac_{2}\left(2at_{\pm }+3\right),\;{\langle {\hat{p}}^{4}\rangle }_{\pm }=\frac{3}{4}+a^{2}+a^{2}c_{2}^{2}+3at_{\pm }-ac_{2}\left(2at_{\pm }+3\right),$$

$$\sigma_{A}^{\left(\pm \right)}=\frac{1}{2}-a^{2}t_{\pm }^{2}+a^{2}+2at_{\pm }+2ac_{2},\;\sigma_{B}^{\left(\pm \right)}=\frac{1}{2}-a^{2}t_{\pm }^{2}+a^{2}+2at_{\pm }-2ac_{2},$$

$$\sigma_{AB}^{\left(\pm \right)}=a^{2}-a^{2}t_{\pm }^{2}-1/2,\;Y_{AB}^{\left(\pm \right)}=2as_{2},$$

where $a={\left|\alpha \right|}^{2}$, $\alpha =\left|\alpha \right|e^{i\phi }$, $t_{\pm }={\left[\tanh\left(a\right)\right]}^{\pm 1}$, $c_{n}=\cos\left(n\phi \right)$, $s_{n}=\sin\left(n\phi \right)$, so that $c_{4}=c_{2}^{2}-s_{2}^{2}$. Hence, we have to minimize the following functions:

$$Z_{\pm }=\frac{1}{4}\left[{\left(1-2a^{2}t_{\pm }^{2}+2a^{2}+4at_{\pm }\right)}^{2}-16a^{2}\right],\;{\left(\Pi_{\sigma }^{\left(4\right)}\right)}_{\pm }=\frac{1}{4}\left[{\left(1-2a^{2}t_{\pm }^{2}+2a^{2}+4at_{\pm }\right)}^{2}-16a^{2}c_{2}^{2}\right],$$

(31) $$\Pi_{\pm }^{\left(4\right)}={\left(3/4+a^{2}+3at_{\pm }+a^{2}c_{2}^{2}\right)}^{2}-a^{2}c_{2}^{2}{\left(2at_{\pm }+3\right)}^{2}.$$

Note that functions $Z_{\pm }$ do not depend on the phase ϕ. Their minimal values (found numerically) are:

$$Z_{+}^{min}=0.21259\;\;\mathrm{for}\;|\alpha |\approx 0.52963,\;Z_{-}^{min}=1.76386\;\;\mathrm{for}\;\left|\alpha \right|\approx 1.03430.$$

The same minimal values are obtained for the products ${\left(\Pi_{\sigma }^{\left(4\right)}\right)}_{\pm }$, if $\cos^{2}\left(2\phi \right)=1$. The following mean values can be found for |α|=0.52963 and $c_{2}=-1$:

$${\langle {\hat{x}}^{2}\rangle }_{+}=0.296176,\;{\langle {\hat{x}}^{4}\rangle }_{+}=0.252876,\;\sigma_{A}^{\left(+\right)}=0.165156,$$

$${\langle {\hat{p}}^{2}\rangle }_{+}=0.857192,\;{\langle {\hat{p}}^{4}\rangle }_{+}=2.021965,\;\sigma_{B}^{\left(+\right)}=1.287187.$$

The minimal values of $\langle {\hat{p}}^{2}\rangle$, $\langle {\hat{p}}^{4}\rangle$, and $\sigma_{B}$ are obtained for $c_{2}=1$. These minimal values are as follows,

$${\langle {\hat{p}}^{2}\rangle }_{+}^{min}=0.221535\;\;\mathrm{for}\;|\alpha |=0.79952,\;{\langle {\hat{p}}^{4}\rangle }_{+}^{min}=0.202769\;\;\mathrm{for}\;\left|\alpha \right|=0.654213,$$

$$\sigma_{B}^{\left(+min\right)}=0.140771\;\;\mathrm{for}\;\left|\alpha \right|=0.626735.$$

The product $\Pi_{\pm }^{\left(4\right)}$, as given by Equation (31), is a quadratic polynomial with respect to the variable $\xi =c_{2}^{2}$. It is easy to verify that the derivative of this polynomial with respect to ξ is negative in the interval 0≤ξ≤1. Consequently, the minimum is achieved at ξ=1. Thus, we have to minimize the function

$$f\left(a\right)=\frac{9}{16}+\frac{9}{2}at_{\pm }-6a^{2}+9a^{2}t_{\pm }^{2}+4a^{4}\left(1-t_{\pm }^{2}\right).$$

Numerical calculations yield

$${\left(\Pi_{+}^{\left(4\right)}\right)}_{min}=0.51057\;\;\mathrm{for}\;a=0.26657,\;{\left(\Pi_{-}^{\left(4\right)}\right)}_{min}=13.07101\;\;\mathrm{for}\;a=0.92917\;.$$

Although ${\left(\Pi_{-}^{\left(4\right)}\right)}_{min}$ is smaller than the value ${\left(\Pi^{\left(4\right)}\right)}_{1}=225/16=14.0625$ in the first Fock state |1〉, the difference is only about 7%. This example shows that, removing the vacuum state, one cannot achieve a small value of $\Pi^{\left(4\right)}$.

**Figure 3:**
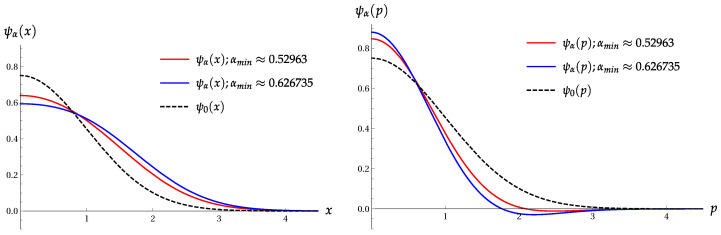
Wave functions of the even coherent states $\psi_{+}\left(x\right)$ and $\psi_{+}\left(p\right)$ (30) for two extremal values of parameter α, as compared with the ground-state wave functions $\psi_{0}\left(x\right)$ and $\psi_{0}\left(p\right)$.

Figure 3 shows the functions $\psi_{+}\left(x\right)$ and $\psi_{+}\left(p\right)$ (30) for two extremal values of parameter α.

### 6.2. Superpositions of Four Coherent States (Orthogonal-Even Coherent States)

For the state (6), we use the following formulas that were obtained in [7,17]:

(32) $$B^{2}={\left\{8e^{-a}\left[\cosh\left(a\right)+\cos\left(a\right)\right]\right\}}^{-1},\;\alpha =\sqrt{a}e^{i\phi },$$

$$\langle {\hat{x}}^{2}\rangle =\langle {\hat{p}}^{2}\rangle =\frac{1}{2}+a\;\frac{\sinh(a)-\sin(a)}{\cosh(a)+\cos(a)},$$

$$\langle {\hat{x}}^{4}\rangle =\langle {\hat{p}}^{4}\rangle =\frac{3}{4}+\frac{a^{2}}{2}\cos\left(4\phi \right)+3\;\frac{a\left[\sinh\left(a\right)-\sin\left(a\right)\right]+a^{2}\left[\cosh\left(a\right)-\cos\left(a\right)\right]/2}{\cosh(a)+\cos(a)}.$$

The minimum of $\langle {\hat{x}}^{4}\rangle$ and $\langle {\hat{p}}^{4}\rangle$ is achieved for cos(4ϕ)=−1. According to [7,17], ${\langle {\hat{x}}^{4}\rangle }_{min}=0.69992$ is achieved at |α|=0.67. Assuming cos(4ϕ)=−1, we obtain the following expression for the variances:

$$\sigma_{A}=\sigma_{B}=\frac{1}{2}+\frac{a^{2}\left[2\sinh\left(a\right)\sin\left(a\right)-\cosh\left(a\right)\cos\left(a\right)-\cos^{2}\left(a\right)\right]}{{\left[\cosh\left(a\right)+\cos\left(a\right)\right]}^{2}}+\frac{2a[\sinh(a)-\sin(a)]}{\cosh(a)+\cos(a)}.$$

**Figure 4:**
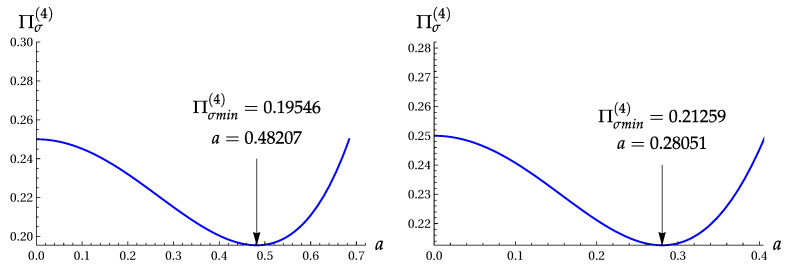
The product $\Pi_{\sigma }^{\left(4\right)}$ versus $a={\left|\alpha \right|}^{2}$. Left: for the OECS (6) with cos(4ϕ)=−1. Right: for the even coherent state (28) with $\cos^{2}\left(2\phi \right)=1$.

The minimum $\sigma_{A}^{\left(min\right)}=0.442$ is achieved for a=0.482, i.e., |α|≈0.694. Hence, ${\left(\Pi_{\sigma }^{\left(4\right)}\right)}_{min}=0.1955$ for the orthogonal-even coherent states. This value is only slightly less than (27), as obtained for the two-term superposition $|\psi_{4}\rangle$. Figure 4 shows the product $\Pi_{\sigma }^{\left(4\right)}$ as function of parameter $a={\left|\alpha \right|}^{2}$ for the OECS with cos(4ϕ)=−1 and the even coherent state with $\cos^{2}\left(2\phi \right)=1$.

Using Equations (6), (29) and (32), the wave function of the OECS with ϕ=π/4 can be written as

$$\psi_{4\alpha }\left(x\right)=\frac{\sqrt{2}\exp\left(-x^{2}/2\right)\left[{\cosh(|\alpha |x)\cos(|\alpha |x)\cos(|\alpha |}^{2}{/2)+\sinh(|\alpha |x)\sin(|\alpha |x)\sin(|\alpha |}^{2}/2)\right]}{\pi^{1/4}\sqrt{{\cosh(|\alpha |}^{2}{)+\cos(|\alpha |}^{2})}}.$$

**Figure 5:**
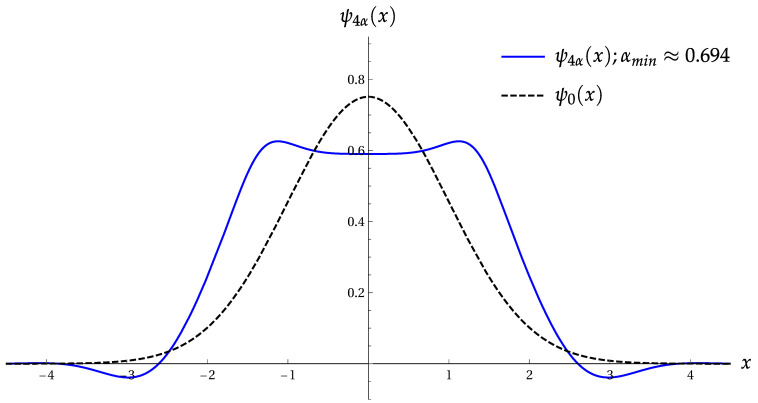
The wave function of the OECS (6) with ϕ=π/4 and |α|=0.694.

The plot of this function for the extremal value |α|=0.694 is shown in Figure 5. It looks rather different from the vacuum function $\psi_{0}\left(x\right)$ and the extremal function $\psi_{4}\left(x\right)$, as shown in Figure 2.

## 7. Sixth Order Products in Two-Term Superpositions of the Fock States

For the Fock states, we have $\langle {\hat{x}}^{3}\rangle =\langle {\hat{p}}^{3}\rangle =0$ and

$$\langle n|{\hat{x}}^{6}|n\rangle =\langle n|{\hat{p}}^{6}n\rangle =\frac{5}{8}\left(2n+1\right)\left(2n^{2}+2n+3\right),\;\langle n|{\hat{x}}^{3}{\hat{p}}^{3}|n\rangle =\frac{3i}{8}\left(6n^{2}+6n+1\right).$$

Therefore, the Fock states are not intelligent for operators ${\hat{x}}^{3}$ and ${\hat{p}}^{3}$ in any sense.

The sixth order moments grow very rapidly with *n*:

$$\langle 0|{\hat{x}}^{6}|0\rangle =15/8,\;\langle 2|{\hat{x}}^{6}|2\rangle =375/8,\;\langle 3|{\hat{x}}^{6}|3\rangle =945/8,\;\langle 4|{\hat{x}}^{6}|4\rangle =1935/8,\;\langle 6|{\hat{x}}^{6}|6\rangle =5655/8.$$

Nonetheless, some simple two-term superpositions of the Fock states $|\psi_{k}\rangle$ of the form (3) can give the values of $\Pi^{\left(6\right)}$ smaller than the vacuum value ${\left(\Pi^{\left(6\right)}\right)}_{vac}=225/64=3.515625$. Note that we have $\langle {\hat{x}}^{3}\rangle =\langle {\hat{p}}^{3}\rangle =0$ for even values of *k*, so that $\Pi^{\left(6\right)}=\Pi_{\sigma }^{\left(6\right)}$ in such cases.

For k=2, we have

〈x^6〉=385+120|β|2+302Re(α*β),〈p^6〉=385+120|β|2−302Re(α*β).

The minimum of the product $\Pi^{\left(6\right)}$ is attained for [Re(α*β)]2=|αβ|2. Subsequently,

$$\Pi^{\left(6\right)}=\frac{9}{64}\left[{\left(5+{120|\beta |}^{2}\right)}^{2}-1800{\left|\beta \right|}^{2}\left(1-{\left|\beta \right|}^{2}\right)\right].$$

The minimum of this function is attained at ${\left|\beta \right|}^{2}=1/54$, yielding ${\left(\Pi^{\left(6\right)}\right)}_{min}=175/64\approx 2.73$, which is smaller than the vacuum value.

A better result can be obtained for k=4, with

(33) 〈x^6〉=〈p^6〉=385+640|β|2+206Re(α*β).

The minimum of (33) is attained for Re(α*β)=−|αβ| and ${\left|\beta \right|}_{min}^{2}=1/2-\sqrt{128/515}\approx 0.00146$. It yields ${\left(\Pi^{\left(6\right)}\right)}_{min}=\left(225/64\right){\left(65-2\sqrt{1030}\right)}^{2}\approx 2.32\approx 0.66\;{\left(\Pi^{\left(6\right)}\right)}_{vac}$. However, this value is higher than the value ${\left(\Pi^{\left(6\right)}\right)}_{min}={\left(1.4765\right)}^{2}\approx 2.18$ that was obtained in [4,5].

For k=6, we have

〈x^6〉=385+1880|β|2+85Re(α*β),〈p^6〉=385+1880|β|2−85Re(α*β).

Now, the minimum of $\Pi^{\left(6\right)}$ is attained for [Re(α*β)]2=|αβ|2. Subsequently,

$$\Pi^{\left(6\right)}=\frac{9}{64}\left[{\left(5+{1880|\beta |}^{2}\right)}^{2}-320{\left|\beta \right|}^{2}\left(1-{\left|\beta \right|}^{2}\right)\right].$$

However, the derivative of this function with respect to ${\left|\beta \right|}^{2}$ is positive at ${\left|\beta \right|}^{2}=0$. Consequently, the state $|\psi_{6}\rangle$ has the values $\Pi^{\left(6\right)}>{\left(\Pi^{\left(6\right)}\right)}_{vac}$ for any β≠0.

## 8. Asymptotic Values of High-Order Products in the Two-Term Fock Superpositions

It is interesting to see the behavior of ${\left(\Pi^{\left(2n\right)}\right)}_{min}$ for n≫1. This can be easily done for the two-Fock superpositions, while using Equations (22) and (23). Coefficients (24) and (25) have the following asymptotic forms:

$${\tilde{\rho }}_{2j}^{\left(2n\right)}=\frac{{\left(2n\right)}^{j}}{\sqrt{(2j)!}}\left[1-\frac{j(j-1)}{2n}\right],\;{\tilde{\nu }}_{2j}^{\left(2n\right)}+1=\frac{{\left(2n\right)}^{2j}}{(2j)!}\left[1+\frac{j}{n}\right],$$

where we neglect corrections of the relative order $n^{-2}$ inside square brackets. Afterwards, Equations (22) and (23) assume the form

$$z_{*}={\left|\beta \right|}_{*}^{2}\approx {\left(2n\right)}^{-2j}\left(2j\right)!,\;f_{min}\approx j^{2}/n,\;\chi_{min}\approx 4j^{2}/n.$$

If *j* is an even number, then $\langle \psi_{2j}|{\hat{x}}^{2n}|\psi_{2j}\rangle =\langle \psi_{2j}|{\hat{p}}^{2n}|\psi_{2j}\rangle$, so that $F^{\left(2n\right)}\equiv {\left(\Pi^{\left(2n\right)}\right)}_{min}/{\left(\Pi^{\left(2n\right)}\right)}_{vac}=f_{min}^{2}$. Therefore, this ratio is minimized for j=2, i.e., in the state $|\psi_{4}\rangle$, which has preferences over other-two term superpositions for n=2 and n=3, as we saw in the preceding sections:

(34) $$F^{\left(2n\right)}=f_{min}^{2}\approx {\left(4/n\right)}^{2},\;n\gg 1.$$

The authors of [4] minimized the mean value of operator ${\hat{x}}^{N}+{\hat{p}}^{N}$ for the state $|\psi_{4}\rangle$ with an arbitrary real coefficient α. We re-write Formula (33) from that paper in a more convenient form:

(35) $$\sqrt{F^{\left(N\right)}}=\frac{u/48}{w+\sqrt{w^{2}-u}},$$

$$w=N^{4}+4N^{3}+20N^{2}+32N+48,\;u=768\left(N^{3}+2N^{2}+4N+3\right).$$

If N≫1, then (35) assumes the form $\sqrt{F^{\left(N\right)}}\approx 8/N$, which coincides with (34) for N=2n. However, it is worth noticing that such a simple asymptotic formula gives accurate enough values of the ratio $F^{\left(N\right)}$ for very big values of *N* only. Note that the authors of [4] interpreted Formula (35) incorrectly, saying that the uncertainty product goes to zero as N→∞. Of course, this was an obvious oversight: although the ratio $F^{\left(N\right)}$ tends to zero as N→∞, the total uncertainty product $\Pi^{\left(N\right)}={\left(\Pi^{\left(N\right)}\right)}_{vac}F^{\left(N\right)}$ remains very big in this limit.

**Figure 6:**
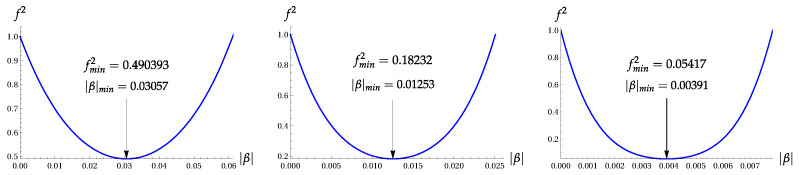
The ratio ${\left(\Pi^{\left(2n\right)}\right)}_{2j}/{\left(\Pi^{\left(2n\right)}\right)}_{vac}\equiv f^{2}$ in the state $|\psi_{4}\rangle$ as function of |β| for ϕ=π. Left: for 2n=8. Middle: for 2n=16. Right: for 2n=32.

**Figure 7:**
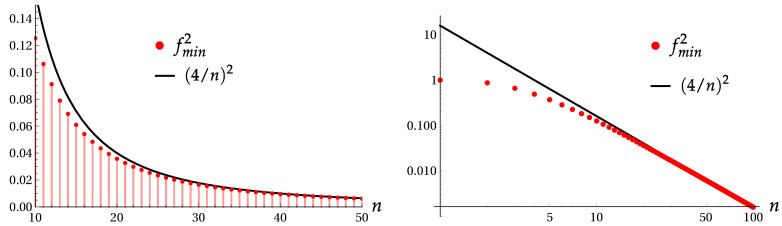
Exact and asymptotic values of the minimal ratio $f_{min}^{2}$ for high values of the power *n* in the usual and logarithmic scales.

Figure 6 shows the ratio ${\left(\Pi^{\left(2n\right)}\right)}_{2j}/{\left(\Pi^{\left(2n\right)}\right)}_{vac}\equiv f^{2}$, calculated using exact Formula (21), as function of |β| for ϕ=π, in the state $|\psi_{4}\rangle$ (i.e., j=2), for different values of power 2n. We see that the asymptotic Formula (34) gives approximate minimal values higher than actual ones. Exact and asymptotic values of $f_{min}^{2}$ are compared in Figure 7.

If *j* is an odd number, then $F^{\left(2n\right)}=\chi_{min}∼4j^{2}/n$. Therefore, the state $|\psi_{4}\rangle$ is the best one among all two-term Fock superpositions from the point of view of the minimization of $\Pi^{\left(2n\right)}$. However, the state $|\psi_{2}\rangle$ is preferable from the point of view of the high-order squeezing: see Section 9. We do not study the variance product $\Pi_{\sigma }^{\left(2n\right)}$ for n≫1, because it is very close to $\Pi^{\left(2n\right)}$: see the end of Section 2.3.

## 9. Significant and Weak High-Order Squeezing

The concept of high-order squeezing was introduced by Hong and Mandel [40]. According to their definition, the state |ψ〉 is squeezed to the 2nth order with respect to $\hat{x}$, if the mean value $\langle \psi |{\left(\Delta \hat{x}\right)}^{2n}|\psi \rangle$ is less than the mean value of ${\left(\Delta \hat{x}\right)}^{2n}$ in the coherent state, i.e., $\langle {\left(\Delta \hat{x}\right)}^{2n}\rangle <\Lambda_{2n}^{vac}\equiv 2^{-n}\left(2n-1\right)!!$. In particular, for n=2 we have the requirement $\langle {\left(\Delta \hat{x}\right)}^{4}\rangle <3/4$. Hong and Mandel showed that the usual squeezed states are squeezed to any even order 2n. There are other definitions of the higher-order squeezing [41,42,43,44], but they are not related directly to the powers of coordinate and momentum operators.

The choice of the coherent (or vacuum) state as the reference one is natural for the usual (second-order) squeezing, because the minimal value of the uncertainty product $\Pi_{\sigma }^{\left(2\right)}$ (as well as $\Pi^{\left(2\right)}$) is achieved in this state. However, minimal values of high-order products are achieved in non-Gaussian states. Therefore, we suggest to say that the *x* variable is significantly squeezed to the 2nth order, if $\langle {\left(\Delta \hat{x}\right)}^{2n}\rangle <\Lambda_{2n}$, where $\Lambda_{2n}=\sqrt{{\left(\Pi^{\left(2n\right)}\right)}_{min}}$. If $\Lambda_{2n}^{vac}>\langle {\left(\Delta \hat{x}\right)}^{2n}\rangle >\Lambda_{2n}$, then the 2nth order squeezing can be considered as weak. In particular, using the results from [4], we can use the following values:

(36) $$\Lambda_{4}\approx 0.7,\;\Lambda_{6}\approx 1.48,\;\Lambda_{8}\approx 4.14,\;\Lambda_{10}\approx 17.96.$$

In addition, we can consider the σ-squeezing of the fourth order, defining its weak form by the inequality $\sigma_{x^{2}}<1/2$, and the significant form by the inequality $\sigma_{x^{2}}<{\left(\Lambda_{\sigma }\right)}_{4}=\sqrt{{\left(\Pi_{\sigma }^{\left(4\right)}\right)}_{min}}\approx 0.44$. Note that, according to our definition, the simultaneous significant high-order squeezing is impossible.

For example, all of the Gaussian states with b=c=d=0 (see Section 2) possess some amount of weak high-order squeezing with respect to *x* for any value a>1 and any value 2n. However, the significant fourth-order squeezing can be achieved for $a>a_{4}=1.035$, and the significant σ-squeezing of the fourth order exists for a>1.066. For higher values of 2n, the minimal value of parameter *a* resulting in the significant squeezing is given by the formula $a_{2n}={\left[F^{\left(2n\right)}\right]}^{-1/(2n)}$, according to Equation (13). This minimal value $a_{2n}$ slowly grows initially with the power 2n, but for 2n≥16 it slowly decays to unity as n→∞. In particular, using the best available values of $\Lambda_{2n}$ from Equation (36), we obtain

$$a_{4}\approx 1.035,\;a_{6}\approx 1.083\left(1.0715\right),\;a_{8}\approx 1.12\left(1.093\right),\;a_{10}\approx 1.105,\;a_{16}\approx 1.112.$$

**Figure 8:**
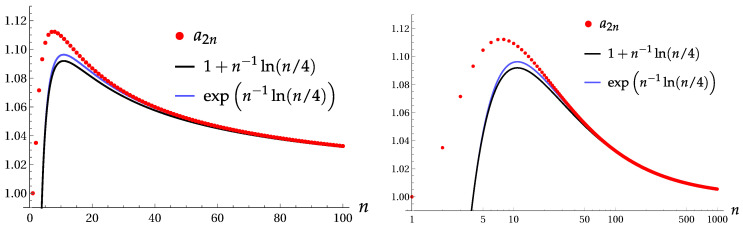
Minimal values $a_{2n}$, when the homogeneous Gaussian state is significantly squeezed to the order 2n, with the normal and logarithmic scales of the horizontal axis.

The values in parentheses for 2n=6 and 2n=8 are obtained using Equation (22), derived for the two-Fock superpositions. For other values of 2n, the results of the two-Fock approximation practically coincide with that of [4]. The asymptotic formula is $a_{2n}\approx \exp\left[n^{-1}\ln\left(n/4\right)\right]\approx 1+n^{-1}\ln\left(n/4\right)$ for n≫4. Therefore, homogeneous Gaussian states with parameter a>1.12 (or $\sigma_{x}<0.45$) are significantly squeezed with respect to the *x*-coordinate to any order 2n. Figure 8 shows minimal values $a_{2n}$, calculated with the aid of Equation (22), as compared with two asymptotic expressions.

**Figure 9:**
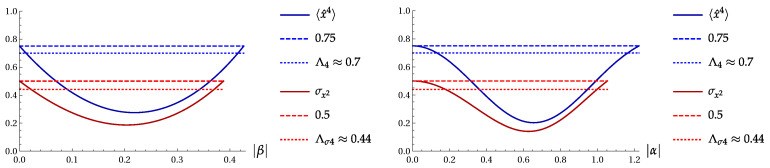
$\langle {\hat{x}}^{4}\rangle$ and $\sigma_{x^{2}}$ as function of parameter |β| for the two-Fock superposition $|\psi_{2}\rangle$ (3) with ϕ=π (left) and as function of parameter |α| for the even coherent state (28) with cos(2ϕ)=−1 (right).

Figure 9 shows $\langle {\hat{x}}^{4}\rangle$ and $\sigma_{x^{2}}$ as function of parameter |β| for the two-Fock superposition $|\psi_{2}\rangle$ (3) with ϕ=π, and as function of parameter |α| for the even coherent state (28) with cos(2ϕ)=−1. Four horizontal lines show the boundaries of weak and significant fourth-order squeezing and σ-squeezing.

**Figure 10:**
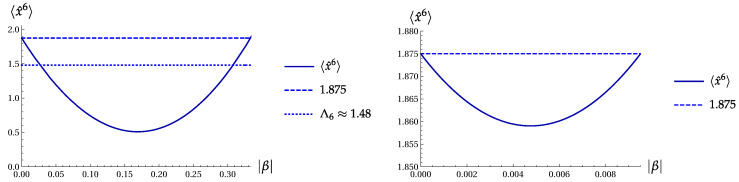
$\langle {\hat{x}}^{6}\rangle$ as function of parameter |β| for the two-Fock superpositions $|\psi_{2}\rangle$ (3) (left) and $|\psi_{6}\rangle$ (right) with ϕ=π.

Figure 10 shows $\langle {\hat{x}}^{6}\rangle$ as function of parameter |β| for the two-Fock superpositions $|\psi_{2}\rangle$ and $|\psi_{6}\rangle$ (3) with ϕ=π. In the case of $|\psi_{2}\rangle$, the minimal value ${\langle {\hat{x}}^{6}\rangle }_{min}=0.510146$ is achieved at |β|=0.169102. For $|\psi_{6}\rangle$, the minimal value ${\langle {\hat{x}}^{6}\rangle }_{min}=1.85904$ is achieved at |β|=0.00475743. In this case, we only observe a very weak sixth-order squeezing.

The Hong–Mandel squeezing of the 4th and 6th order in the even coherent states was studied, e.g., in [45,46,47]. Arbitrary values of 2n were considered in [48]. The most general superpositions of two coherent states were considered in [49,50] in connection with the 4th order squeezing, and in [51,52] in connection with the 2nth order Hong–Mandel squeezing. The enhancement of the 2nth order Hong–Mandel squeezing in superpositions of even coherent states and the vacuum state was demonstrated in [53]. Two-mode generalizations of even/odd coherent states were studied in [54,55].

The high-order Hong–Mandel squeezing in the Gaussian mixed states (squeezed thermal states) was studied, e.g., in paper [56]. Squeezed number states were studied from this point of view in [57]. Multiphoton non-Gaussian states were discussed in [58]. Paper [59] showed the existence of high-order squeezing in specific four-photon states. Combinations of squeezed and number states were considered in [60]. Two-mode squeezed states were considered in [61]. Higher-order squeezing in the photon-added coherent states and their generalizations was studied in [62,63]. “Semi-coherent” states and their generalizations were investigated in this connection in [64,65]. The binomial states and their generalizations were considered in [66,67]. The generation of high-order squeezing in multiphoton processes was studied in [68].

**Figure 11:**
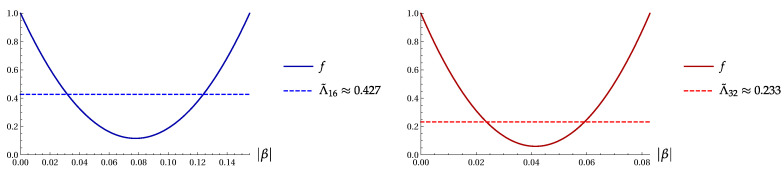
The squeezing coefficient of the 2nth order $f=\langle \psi_{2}|{\hat{x}}^{\left(2n\right)}|\psi_{2}\rangle /\langle 0|{\hat{x}}^{\left(2n\right)}|0\rangle$ as function of parameter |β| for the two-Fock superposition $|\psi_{2}\rangle$ (3) with ϕ=π. Left: 2n=16. Right: 2n=32.

The concept of significant squeezing can be especially important for n≫1, when the significant squeezing boundary $\Lambda_{2n}$ is approximately n/4 times lower than the vacuum (Hong–Mandel) boundary $\Lambda_{2n}^{vac}$. Figure 11 shows the ratio $\langle \psi_{2}|{\hat{x}}^{\left(2n\right)}|\psi_{2}\rangle /\langle 0|{\hat{x}}^{\left(2n\right)}|0\rangle \equiv f$ as function of parameter |β| for the two-Fock superposition $|\psi_{2}\rangle$ (3) with ϕ=π. Function *f* is given by Equation (21) with j=1. The normalized significant squeezing level ${\tilde{\Lambda }}_{2n}=f_{min}$ is calculated while using the exact Formula (22) with j=2.

The high-order σ-squeezing practically coincides with the simple high-order squeezing for n≫1, because $\nu_{2j}^{\left(2n\right)}/{\left[\nu_{2j}^{\left(n\right)}\right]}^{2}∼\left(2j\right)!2^{n}{\left(n/2\right)}^{-2j}\gg 1$ and $\rho_{2j}^{\left(2n\right)}/{\left[\rho_{2j}^{\left(n\right)}\right]}^{2}∼\sqrt{(2j)!}2^{n}{\left(n/2\right)}^{-j}\gg 1$ in this case. However, the boundaries of two kinds of squeezing are quite different if *n* is not very big.

## 10. Conclusions

Let us emphasize the main results of our study. We have analyzed the high-order moments of $\hat{x}$ and $\hat{p}$ operators in the most general pure inhomogeneous Gaussian (squeezed) states: see, e.g., Formulas (12) and (14). We have found that the minimal product of the fourth order variances $\Pi_{\sigma }^{\left(4\right)}$ for this specific family of quantum states is attained for all of the uncorrelated vacuum squeezed states: see Equation (15). However, this product can be diminished by more than 20% in the two-Fock superpositions $|\psi_{4}\rangle$ (see Equation (27)) and in the compass (four-photon) states [see Section 6.2].

We have shown that all of the Fock states are Robertson–Schrödinger intelligent states (RSIS) for the pair of operators ${\hat{x}}^{2}$ and ${\hat{p}}^{2}$, although they are not ordinary intelligent states for this pair. Additionally, we have discovered that the only Gaussian ordinary intelligent state for the pair ${\hat{x}}^{2},{\hat{p}}^{2}$ is the correlated coherent state with the correlation coefficient $r=\pm 1/\sqrt{2}$.

We have analyzed in detail the uncertainty products of the 4th and 6th orders in the two-Fock and three-Fock superpositions. In this connection, we have found new explicit expressions for the mean values and matrix elements between arbitrary Fock states for arbitrary powers of operator $\hat{x}$ (as well as for $\hat{p}$): see Equations (A7) and (A11) in Appendix B. Using these expressions, we have obtained the asymptotic Formula (34) for the uncertainty product $\Pi^{\left(2n\right)}$ with n≫1 in the two-Fock superpositions, clarifying the earlier results of paper [4].

We have introduced the new concept of weak and significant high-order squeezing. It was illustrated in the examples of Gaussian states and two-Fock superpositions. The distinguished role of the superposition of the vacuum and the fourth Fock state was demonstrated.

One of the most impressive results is that very small deformations of the vacuum state can result in big changes of such quantities as the high-order uncertainty product or high-order squeezing. This is an indication to the insufficiency of the concept of quantum “fidelity” (the scalar product between two states in the case of pure quantum states) as a measure of distinguishability between quantum states in certain physical situations. See, in this connection, e.g., earlier papers [69,70,71,72,73,74,75].

Several interesting questions and problems arise in connection with our study. For example, is it possible to obtain more precise limits for the high-order uncertainty products for several degrees of freedom, generalizing known results for the products of variances [2,29,76,77,78,79,80,81,82]? Probably, applications to the problem of intensity-difference squeezing [83,84] can be found. Or, how to generalize our results to the case of mixed quantum states with the fixed value of quantum purity (see, e.g., studies [85,86] for the second-order uncertainty products)? It would be interesting to verify the new relations in experiments, for example, using the scheme of measuring high-order moments of canonical variables proposed in [87] (some experiments related to uncertainty relations for the second-order moments were performed in [88]; experimental verifications of the uncertainty relations for the angular momentum operator and other quantum systems in finite-dimensional Hilbert spaces were reported in [89,90,91,92,93,94]). Other interesting areas, where high-order uncertainty relations can be useful, include entanglement, steerability, and Bell nonlocality (see, e.g., studies [95,96,97]). However, all of these problems need separate investigations.

## Appendix A. Details of Calculations for the Gaussian States

To derive the first formula in Equation (12), we start from the expressions

$$\langle {\hat{x}}^{n}\rangle ={\left(\frac{a}{\pi }\right)}^{1/2}\int_{-\infty }^{\infty }x^{n}\exp\left[-a{\left(x-\frac{c}{a}\right)}^{2}\right]dx={\left(\pi a^{n}\right)}^{-1/2}\int_{-\infty }^{\infty }y^{n}\exp\left[-{\left(y-\frac{c}{\sqrt{a}}\right)}^{2}\right]dy.$$

The second integral is well known: see, e.g., Equation 3.462.4 in [98]. It results in the formula

(A1) $$\langle {\hat{x}}^{n}\rangle ={\left(2i\sqrt{a}\right)}^{-n}H_{n}\left(ic/\sqrt{a}\right),$$

where $H_{n}\left(z\right)$ is the Hermite polynomial, defined according to the formula

(A2) $$H_{n}\left(y\right)={\left(-1\right)}^{n}e^{y^{2}}\left(\frac{d^{n}}{dy^{n}}\right)e^{-y^{2}}.$$

Using the relations (11), we obtain from (A1) the first equality in Equation (12). Then, the second equality in (12) is the consequence of the fact that the Fourier transform of any Gaussian function is a Gaussian function again. A formula similar to (A1) for the powers of linear momentum has the form

(A3) $$\langle {\hat{p}}^{n}\rangle =i^{-n}{\left[\left(a^{2}+b^{2}\right)/\left(4a\right)\right]}^{n/2}H_{n}\left(\frac{i(ad-bc)}{\sqrt{a\left(a^{2}+b^{2}\right)}}\right).$$

In particular, the special cases of (A1) and (A3) for n=1 and n=2 yield the formulas

(A4) $$\langle \hat{x}\rangle =c/a,\;\sigma_{x}={\left(2a\right)}^{-1},\;\langle \hat{p}\rangle =\left(ad-bc\right)/a,\;\sigma_{p}=\left(a^{2}+b^{2}\right)/\left(2a\right),$$

confirming the known fact that all moments in the Gaussian states are determined by the first-order mean values and variances. We can write (A1) and (A3) also in terms of the complex numbers α=a+ib and β=c+id:

(A5) 〈x^n〉=2iRe(α)−nHniRe(β)Re(α),〈p^n〉=i−n|α|24Re(α)n/2HniIm(α*β)|α|2Re(α).

A simple formula for the mean value $\langle {\hat{x}}^{n}{\hat{p}}^{n}\rangle$ can be obtained, if $\langle \hat{x}\rangle =\langle \hat{p}\rangle =0$. Indeed, using the relation $-\alpha^{*}=\alpha -2a$ and the definition (A2), one can see that

$$\langle {\hat{x}}^{n}{\hat{p}}^{n}\rangle =i^{n}\sqrt{a/\pi }{\left(\alpha /2\right)}^{n/2}\int_{-\infty }^{\infty }x^{n}e^{-ax^{2}}H_{n}\left(x\sqrt{\alpha /2}\right).$$

Then formula 7.385.2 from [98] yields Equation (14).

## Appendix B. Explicit Expressions for the Coefficients of Nth Order Moments in the Two-Term Even Fock Superpositions

For relatively small values of integers *n* and *j*, the coefficients $\nu_{2j}^{\left(2n\right)}$ and $\rho_{2j}^{\left(2n\right)}$ (19) can be calculated, using the standard bra-ket technique. However, for big values of these indexes it is better to use integrals containing the wave functions. Then, coefficient $\rho_{2j}^{\left(2n\right)}$ equals

(A6) $$\rho_{2j}^{\left(2n\right)}=\frac{2^{1-j}}{\sqrt{\pi (2j)!}}\int_{0}^{\infty }x^{2n}\exp\left(-x^{2}\right)H_{2j}\left(x\right)dx.$$

This integral is a special case of formula 7.376.2 from [98] (we have changed the notation):

$$\int_{0}^{\infty }e^{-sx^{2}}x^{g}H_{2j}\left(x\right)dx=\frac{{\left(-1\right)}^{j}2^{2j-1}}{\sqrt{\pi }s^{(g+1)/2}}\Gamma \left[\left(g+1\right)/2\right]\Gamma \left(j+1/2\right)F\left(-j,(g+1)/2;1/2;1/s\right).$$

Here Γ(z) is the Gamma-function and F(a,b;c;z) is the Gauss hypergeometric function. The next step is to use formula 8.962.1 from [98] and to rewrite the hypergeometric function with the first negative integral parameter a=−j in terms of the Jacobi polynomial:

$${\left(-1\right)}^{j}F\left(-j,(g+1)/2;1/2;1/s\right)=\frac{j!\Gamma (1/2)}{\Gamma (j+1/2)}P_{j}^{\left(g/2-j,-1/2\right)}\left(2/s-1\right).$$

Consequently,

$$\int_{0}^{\infty }e^{-sx^{2}}x^{g}H_{2j}\left(x\right)dx=\frac{2^{2j-1}j!}{s^{(g+1)/2}}\Gamma \left[\left(g+1\right)/2\right]P_{j}^{\left(g/2-j,-1/2\right)}\left(2/s-1\right).$$

In the case of (A6), we have s=1 and g=2n. Then, using the formulas

$$P_{j}^{\left(a,b\right)}\left(1\right)=\frac{\Gamma (j+a+1)}{j!\Gamma (a+1)},\;\Gamma \left(n+1/2\right)=\sqrt{\pi }2^{-n}\left(2n-1\right)!!,$$

we finally obtain the expressions

(A7) $$\rho_{2j}^{\left(2n\right)}=\frac{2^{j-n}n!\left(2n-1\right)!!}{\left(n-j\right)!\sqrt{(2j)!}}=\frac{2^{j-2n}\left(2n\right)!}{\left(n-j\right)!\sqrt{(2j)!}},$$

which clearly show that $\rho_{2j}^{\left(2n\right)}=0$ if n<j. An immediate consequence of (A7) is Equation (24) for the normalized coefficient ${\tilde{\rho }}_{2j}^{\left(2n\right)}$.

The coefficient $\nu_{2j}^{\left(2n\right)}$ is given by the integral

(A8) $$\nu_{2j}^{\left(2n\right)}=2{\left(\sqrt{\pi }\;2^{2j}\left(2j\right)!\right)}^{-1}\int_{0}^{\infty }x^{2n}\exp\left(-x^{2}\right){\left[H_{2j}\left(x\right)\right]}^{2}dx.$$

Here we can use Equation 2.20.16.15 from [99] in the form

$$\int_{0}^{\infty }x^{2g}e^{-x^{2}}H_{2j}\left(x\right)H_{2m}\left(x\right)dx=\frac{2^{2j+2m-2g-1}\sqrt{\pi }\Gamma \left(1+2g\right)}{\Gamma (1+g-j-m)}F\left(-2j,-2m;1+g-j-m;1/2\right).$$

Therefore,

(A9) $$\nu_{2j}^{\left(2n\right)}=\frac{4^{j-n}\left(2n\right)!}{(2j)!(n-2j)!}F\left(-2j,-2j;1+n-2j;1/2\right).$$

However, this formula is good for n≥2j. If n<2j, then we can use the identity 9.131.2 from [98]:

(A10) $$\begin{array}{ccc} F(a,b;c;z) & = & \frac{\Gamma (c)\Gamma (c-a-b)}{\Gamma (c-a)\Gamma (c-b)}F\left(a,b;a+b-c+1;1-z\right)\\ & & +{\left(1-z\right)}^{c-a-b}\frac{\Gamma (c)\Gamma (a+b-c)}{\Gamma (a)\Gamma (b)}F\left(c-a,c-b;c-a-b+1;1-z\right). \end{array}$$

For a=b=−2j, the second term in (A10) turns into zero (we assume initially that *c* is not an integer), so that we arrive at the formula

(A11) $$\nu_{2j}^{\left(2n\right)}=\frac{4^{j-n}\left(2n\right)!\left(n+2j\right)!}{\left(2j\right)!{\left(n!\right)}^{2}}F\left(-2j,-2j;-n-2j;1/2\right),$$

which has no restrictions on the values of *n* and *j*. One can check the validity of Formula (A11) in the case of n=0. Using the formula ${}_{1}F_{0}\left(a;z\right)={\left(1-z\right)}^{-a}$, one obtains $\nu_{2j}^{\left(0\right)}=1$, i.e., the correct normalization of the wave functions 〈x|2j〉. For j=0, Formula (A11) yields the correct value of coefficient $\mu^{\left(2n\right)}$. The consequence of (A11) is Equation (25) for the normalized coefficient ${\tilde{\rho }}_{2j}^{\left(2n\right)}$.
